# Supplementary material for: TGF-β family ligands exhibit distinct signalling dynamics that are driven by receptor localisation
Source: J Cell Sci. 2019 Jul 15;132(14):jcs234039. doi: 10.1242/jcs.234039 (PMC6679586; doi:10.1242/jcs.234039)
Supplement: Supplementary information [file joces-132-234039-s1.pdf]

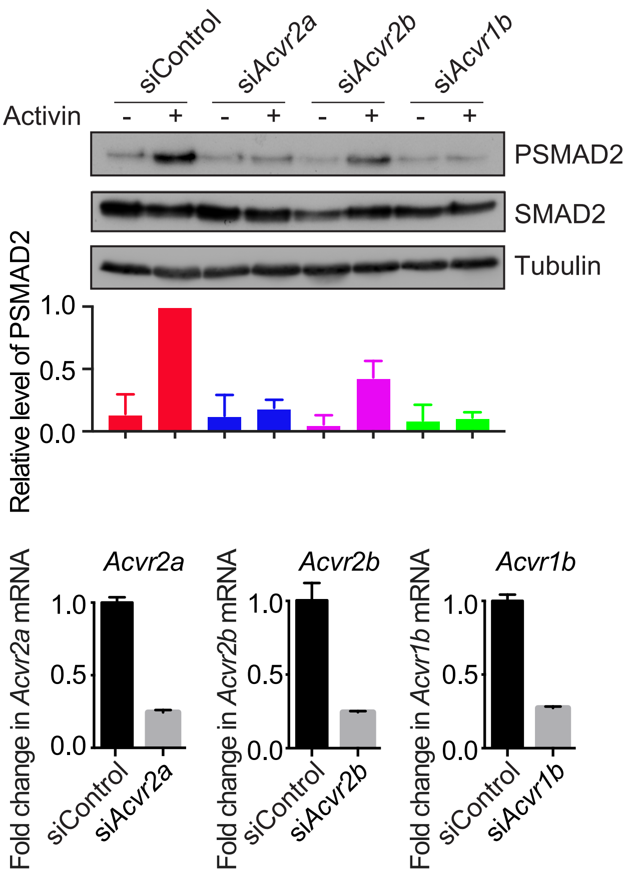

Figure S1. Characterisation of activin receptors in P19 cells.
P19s were transfected with siRNAs against *Acvr2a, Acvr2b* or *Acvr1b* or a non-targeting control (siControl), then treated or not with activin for 1 hr. Western blotting for PSMAD2, SMAD2 and tubulin as a loading control was performed. Quantifications are the means±s.d. of densitometry measurements from two independent experiments, normalised to measurements in siControl cells treated with activin for 1 hr. Below, the extent of knockdown was determined by qPCR. Shown are the normalised means±s.d. from two independent experiments, expressed as fold change in mRNA level relative to non-targeting controls.

Journal of Cell Science • Supplementary information

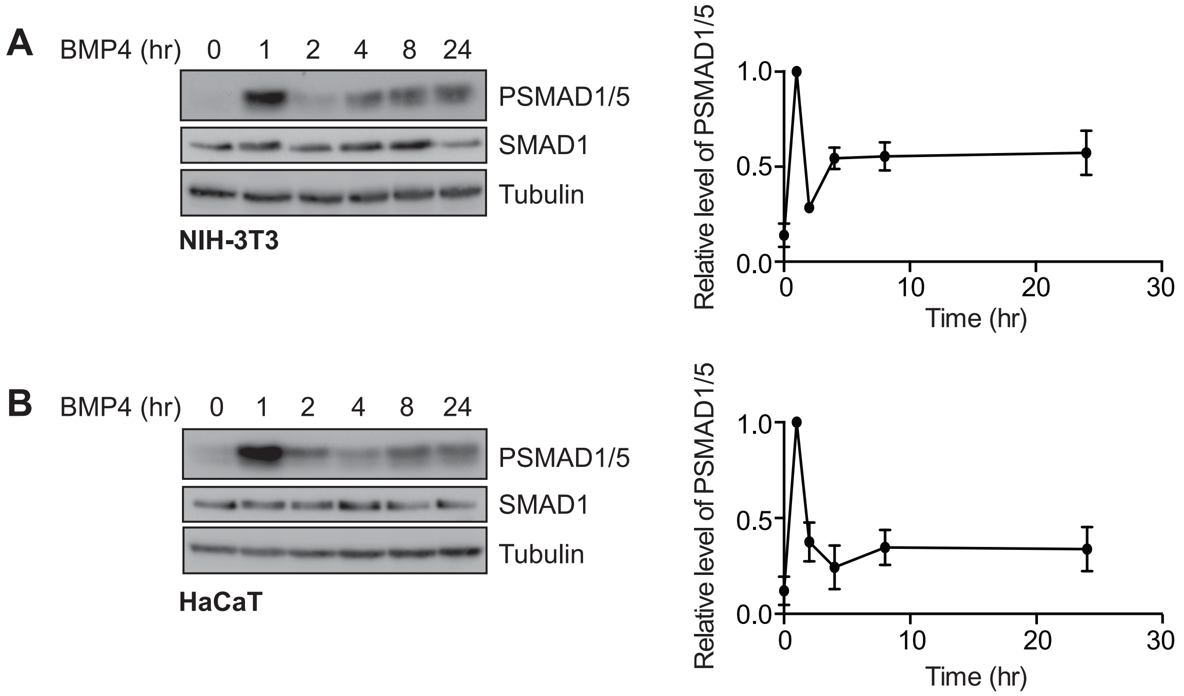

Figure S2. BMP4 exhibits oscillatory signalling in NIH-3T3 cells and in HaCaTs. (**A**) NIH-3T3s or (**B**) HaCaTs were treated with BMP4 for the times indicated. Western blotting for PSMAD1/5, SMAD1 and tubulin as a loading control was performed. Quantifications are the means±s.d. of densitometry measurements from three independent experiments, normalised to measurements in cells treated with BMP4 for 1 hr.

Journal of Cell Science • Supplementary information

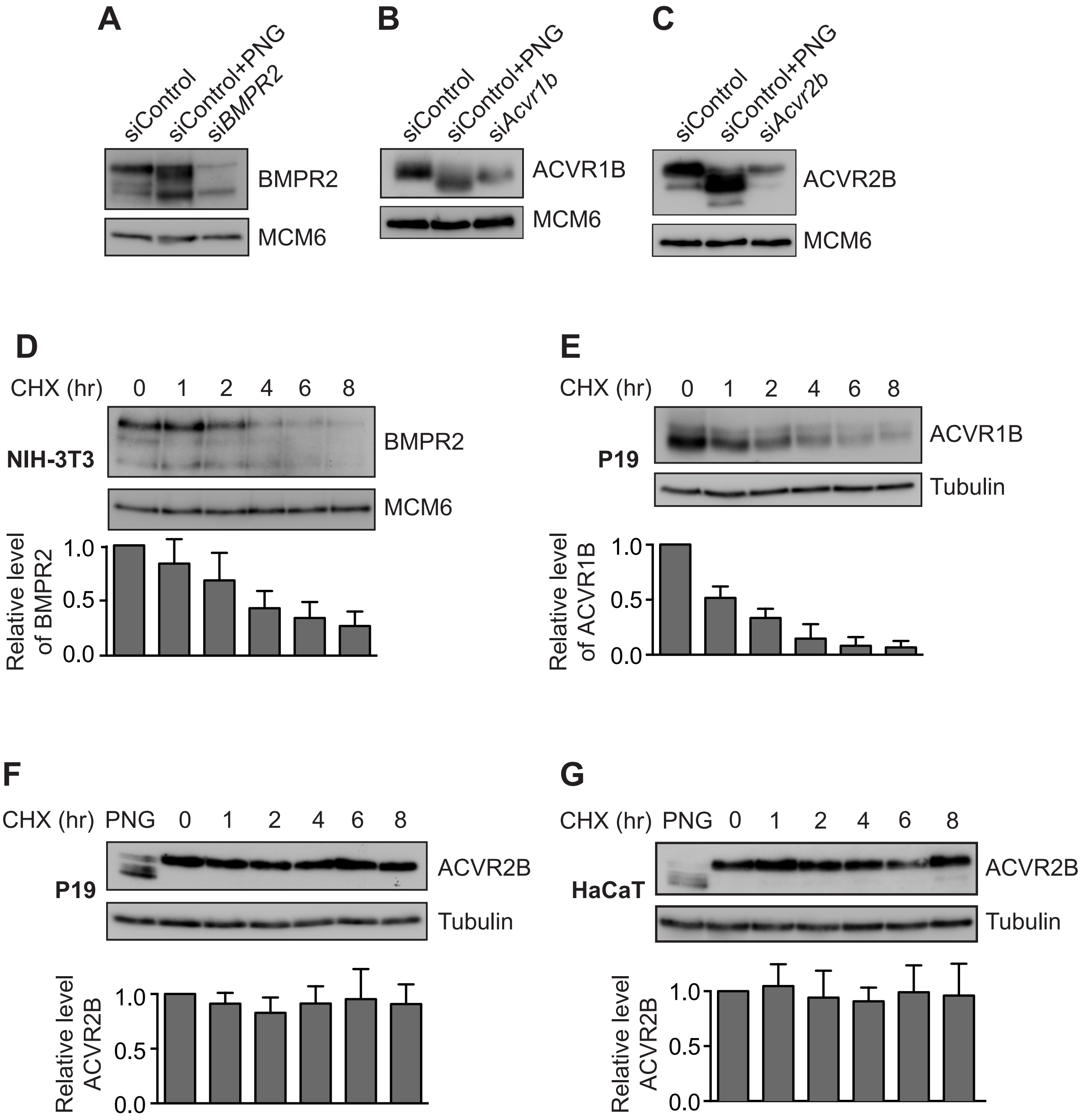

Figure S3. Characterisation of receptor stabilities.
(**A**) MDA-MB-231 cells were transfected with a control non-targeting siRNA (siControl) or siRNAs against BMPR2. (**B** and **C**) P19 cells were transfected with a control non-targeting siRNA (siControl) or siRNAs against *Acvr1b* (B) or *Acvr2B* (C). Lysates were treated or not with PNG. Western blotting for BMPR2, ACVR1B, ACVR2B and MCM6 as a loading control was performed. (**D-G**) NIH-3T3, P19 cells or HaCaTs as indicated were treated with cycloheximide (CHX) for the times indicated. Lysates were treated or not with PNGase (PNG). Western blotting for BMPR2, ACVR1B, ACVR2B and tubulin or MCM6 as a loading control was performed. In all cases, quantifications are the means±s.d. of densitometry measurements from three independent experiments normalised to levels in untreated cells.

Journal of Cell Science • Supplementary information

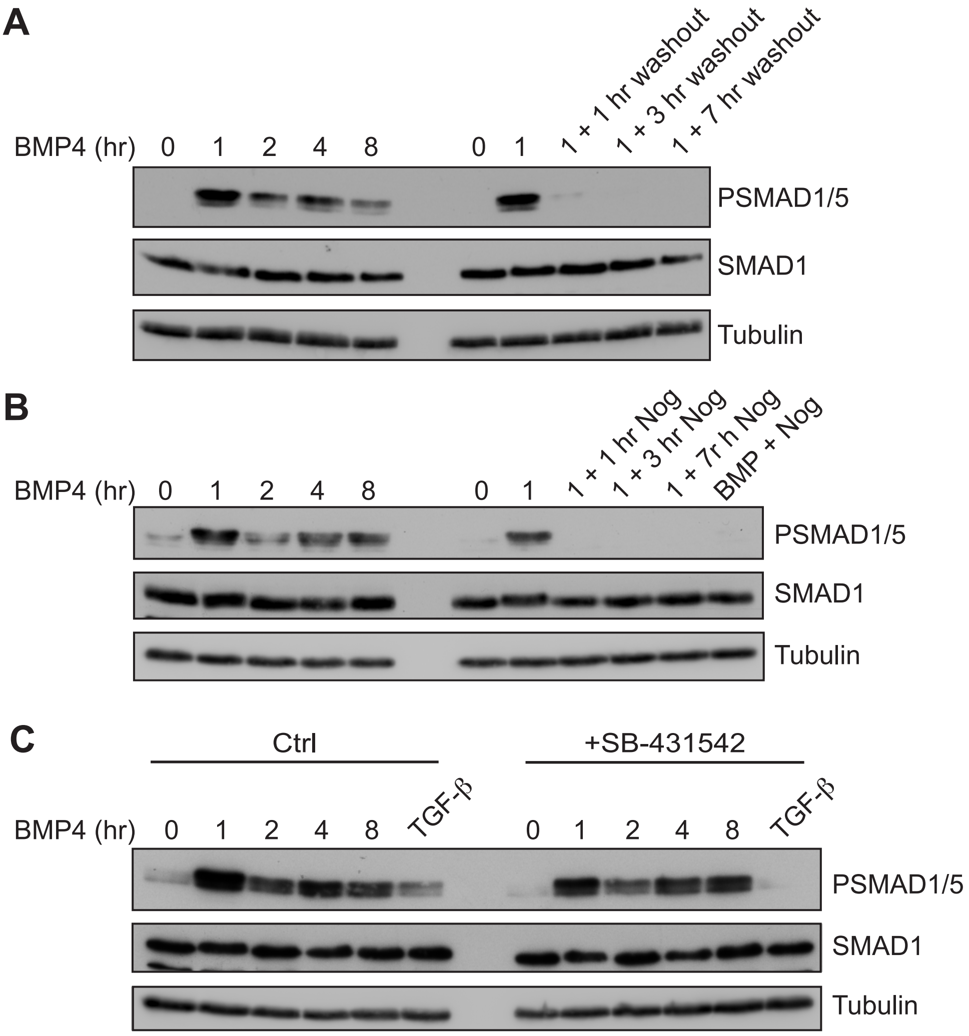

Figure S4. The BMP4 oscillation requires persistent exposure to BMP4 and is not mediated indirectly via SMAD2/3 signalling.

(**A**) NIH-3T3 cells were treated with BMP4 for the times indicated, or for 1 hr with BMP4, before washout and incubation for the times indicated. (**B**) NIH-3T3s were treated for BMP4 for the times indicated, or for 1 hr with BMP4 before addition of noggin for the times indicated. In the final lane, BMP4 and noggin were added simultaneously and cells incubated for 1 hr. (**C**) NIH-3T3 cells were stimulated with BMP4 for the times indicated or with TGF-β for 1 hr, in the absence (Ctrl) or presence (+SB) of SB-431542. Western blotting for PSMAD1/5, SMAD1 or tubulin as a loading control was performed. Representative blots from two independent experiments are shown.

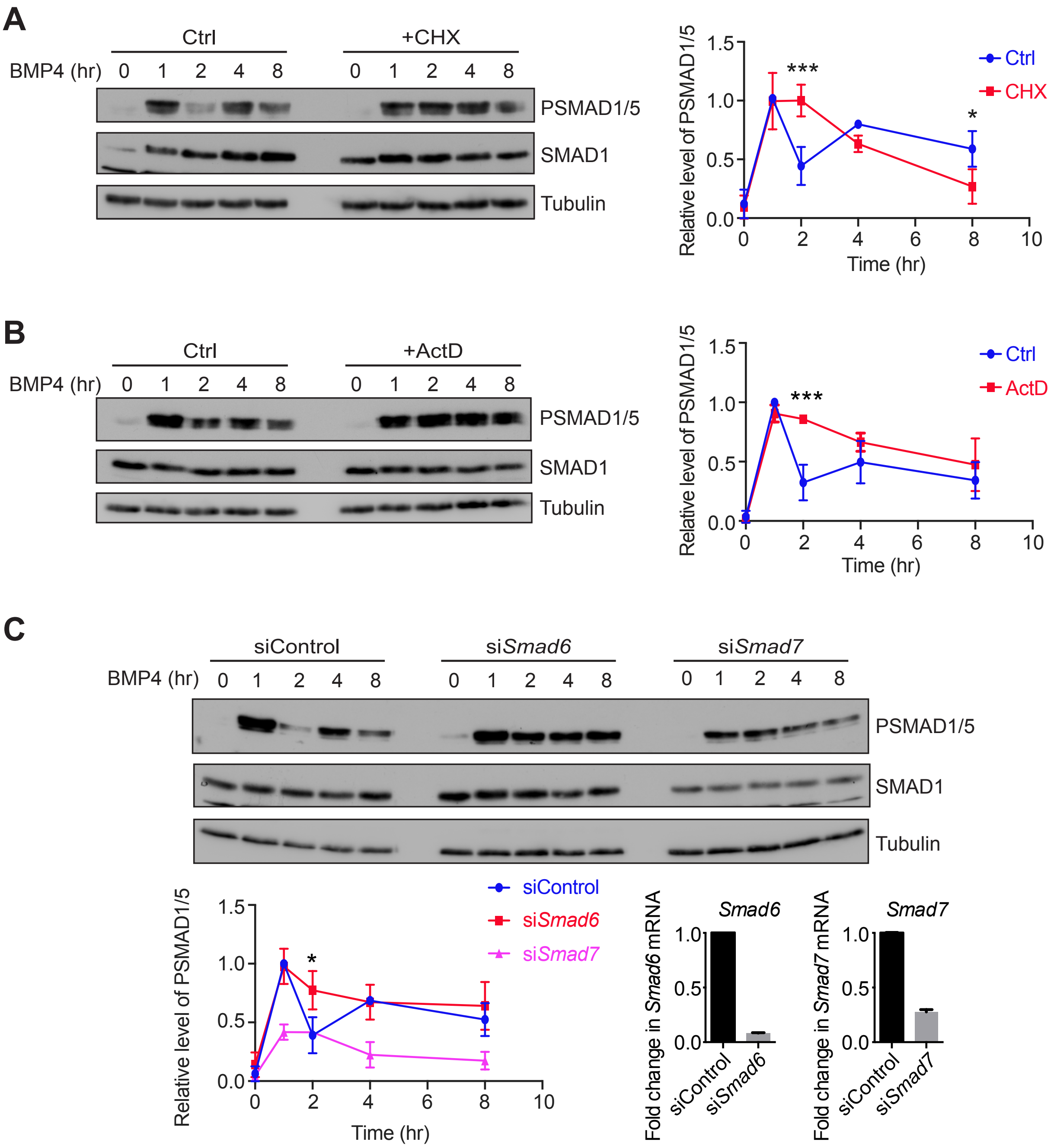

Figure S5. The BMP4 oscillation requires new protein synthesis.
(**A**) NIH-3T3 cells were pre-treated or not with Cycloheximide (CHX) for 5 mins, followed by BMP4 for the times indicated. (**B**) NIH-3T3 cells were pre-treated or not with Actinomycin D (Act D) for 5 mins, followed by BMP4 for the times indicated. In both cases, Western blotting for PSMAD1/5, SMAD1 and tubulin as a loading control was performed. Quantifications are the means±s.d. of densitometry measurements from three independent experiments, normalised to measurements in Ctrl cells treated with BMP4 for 1 hr. *** indicates $p<0.0005$ (**C**) NIH-3T3 cells were transfected with a non-targeting control siRNA (siControl) or siRNAs against *Smad6* or *Smad7* and stimulated with BMP4 for the times indicated. Western blotting for PSMAD1/5, SMAD1 and tubulin as a loading control was performed. Quantifications are the means±s.d. of densitometry measurements from three independent experiments, normalised to measurements in siControl cells treated with BMP4 for 1 hr. * indicates $p<0.05$. Below right, the extent of knockdown was determined by qPCR. Shown are the normalised means±s.d. from two independent experiments, expressed as fold change in mRNA level relative to non-targeting controls.

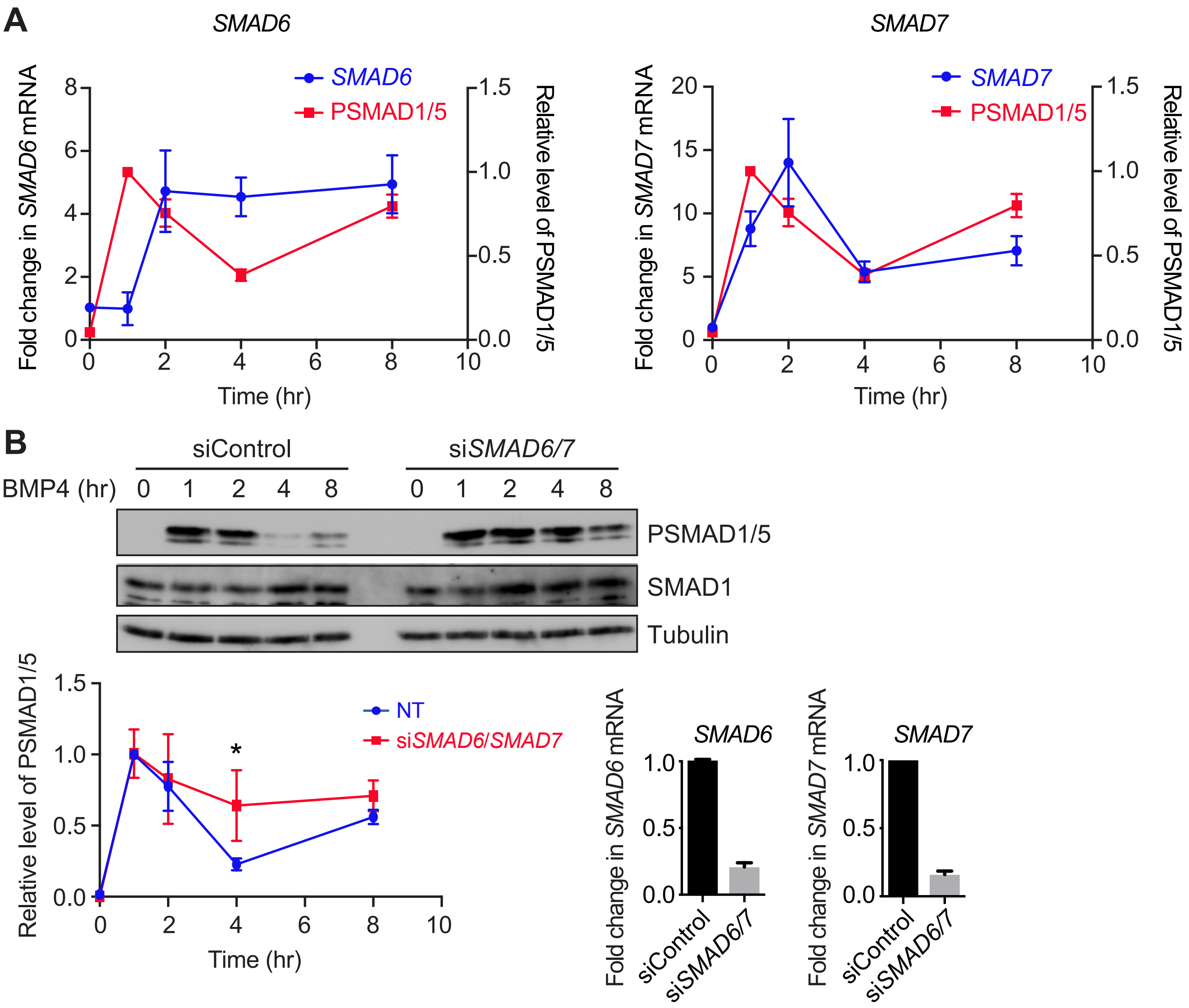

Figure S6. The BMP4 oscillation requires SMAD6/SMAD7 in MDA-MB-231 cells.
(**A**) MDA-MB-231 cells were treated with BMP4 for the times indicated. Levels of *SMAD6* and *SMAD7* mRNA were assayed by qPCR. Shown are the normalised averages and SDs from three independent experiments, expressed as fold change in mRNA level relative to untreated cells. The PSMAD1/5 levels are from the data shown in Fig. 1A. (**B**) MDA-MB-231 cells were transfected with non-targeting control siRNAs (siControl) or siRNA SMARTpools targeting *SMAD6* and *SMAD7*, and were then treated with BMP4 for the times indicated. Western blotting for PSMAD1/5, SMAD1 and tubulin was performed. Quantifications are the means±s.d. of densitometry measurements from three independent experiments, normalised to measurements in siControl cells treated with BMP4 for 1 hr. * indicates $p<0.05$. The extent of knockdown was determined by qPCR. Shown are the normalised means±s.d. from three independent experiments, expressed as fold change in mRNA level relative to non-targeting controls.

Journal of Cell Science • Supplementary information

Table S1

qPCR primers for expression analysis

Target gene	Sequence
Mouse *Smad6* fwd	ATTCTCGGCTGTCTCCTCCT
Mouse *Smad6* rev	GGAGACATGCTGGCATCTGA
Human *SMAD6* fwd	CTGCAACCCCTACCACTTCA
Human *SMAD6* rev	ACATGCTGGCGTCTGAGAAT
Mouse *Smad7* fwd	CCCCCGGCTGAGAGGCTCAT
Mouse *Smad7* rev	CACCTGCTGCCAGTCTGCCC
Human *SMAD7* fwd	CTTAGCCGACTCTGCGAACT
Human *SMAD7*rev	CCAGGCTCCAGAAGAAGTTG
Mouse *Acvr2a* fwd	TCCTACTCAAGACCCAGGACC
Mouse *Acvr2a* rev	TGGGCTTTCCAGACACAACC
Mouse *Acvr2b* fwd	CTACGACAGGCAGGAGTGTG
Mouse *Acvr2b* rev	TGGCTCGTACGTGACTTCTG
Mouse *Acvr1b* fwd	ATCCAGGCTCTGCTGTGTGCG
Mouse *Acvr1b* rev	ACGTACATGGTGCTCCACGCC
Human *GAPDH* fwd	CTTCAACAGCGACACCCACT
Human *GAPDH* rev	GTGGTCCAGGGGTCTTACTC
Mouse *Gapdh* fwd	TCTTGTGCAGTCCCAGCCT
Mouse *Gapdh* rev	CAATATGGCCAAATCCGTTCA

Table S2

siRNAs

Target gene	Catalogue number	Type
Human *SMAD6*	J-015362-05	ON-TARGETplus™ individual duplexes as a pool
	J-015362-06	
	J-015362-07	
	J-015362-08	
Human *SMAD7*	D-020068-01	siGENOME individual duplexes as a pool
	D-020068-02	
	D-020068-03	
	D-020068-04	
Mouse *Smad6*	M-040948-00	siGenome SMARTPool

Journal of Cell Science • Supplementary information

Mouse *Smad7*	M-047242-01	siGenome SMARTPool
Mouse *Acvr1b*	M-043507-01	siGenome SMARTPool
Mouse *Avcr2a*	L-040676-00	ON-TARGETplus™ SMARTPool
Mouse *Acvr2b*	L-040629-00	ON-TARGETplus™ SMARTPool
Human *BMPR2*	D-005309-03	siGENOME individual duplexes as a pool
	D-005309-04	
	D-005309-05	
	D-005309-07	
Non-targeting control (siControl)	D-001206-13-20	siGENOME Non-Targeting siRNA Pool #1

Table S3

Parameters used to model the behaviour of the I-SMADs

Parameter	Value
k_{synbas}^{Ri}	0
k_{syn}^{Ri}	4
k_{deg}^{Ri}	1
K_{Si}	0.02
k_{syn}^{Si}	1
k_{deg}^{Si}	1

Table S4

Key parameters used to model the behaviour of each ligand, with key parameter changes indicated in bold.

Parameter	Value			
Model	BMP4	Activin (HaCaT)	Activin (P19s)	TGF-β
Ligand in ng/ml	20	20	20	**2**
$TSca$	1	0.5	0.5	2
k'_{Toff}	2	2	2	2
k'_T	0.2	0.2	0.2	**100**
D	2	2	2	**4**
k'_{cc}	**0.05**	0.35	0.35	0.35
k_d	1	0.67	0.67	**0.32**
k'^{bas}_{synT}	0	0	**0.8**	0
K_{SBI}	0.001	0.001	0.001	**0.196565**
Y/N Feedback	**1**	0	0	0

Journal of Cell Science • Supplementary information
